# Predictive value of lactate dehydrogenase for *Mycoplasma pneumoniae* necrotizing pneumonia in children based on decision curve analysis and dose–response analysis

**DOI:** 10.1038/s41598-024-60359-1

**Published:** 2024-04-29

**Authors:** Ren Yanhong, Zhao Shuai, Chen Dan, Sun Xiaomin

**Affiliations:** 1 Respiratory Department, Children’s Hospital Affiliated to Zhengzhou University, Henan Children’s Hospital Zhengzhou Children’s Hospital, Zhengzhou, 450018 Henan China; 2 Henan International Joint Laboratory for Infectious Diseases in Children, Children’s Hospital Affiliated to Zhengzhou University, Henan Children’s Hospital Zhengzhou Children’s Hospital, Zhengzhou, 450018 Henan China

**Keywords:** Biomarkers, Medical research

## Abstract

Mycoplasma pneumoniae necrotizing pneumonia (MPNP) has a long and severe disease course, which seriously threatens to jeopardize patients' lives and health. Early prediction is essential for good recovery and prognosis. In the present study, we retrospect 128 children with MPNP and 118 children with *Mycoplasma pneumonia*e pneumonia combined with pulmonary consolidation to explore the predictive value of lactate dehydrogenase (LDH) in children with MPNP by propensity score matching method, multiple logistic regression analysis, dose–response analysis and decision curve analysis. The WBC count, PLT count and percentage of neutrophils were significantly higher in necrosis group than consolidation group. The serum CRP, PCT, ESR, D-D, FIB, ALT, LDH, IgG and IgM were significantly higher in necrosis group. Compared to consolidation group, necrosis group is more severe in chest pain and dyspnea. Multivariate logistic regression analysis showed that duration of LDH levels, high fever, d-dimer, and fibrinogen were independent predictive factors for the incidence of MPNP. Restricted cubic spline analysis showed that a non-linear dose–response relationship between the continuous changes of LDH level and the incidence of MPNP. Decision curve analysis revealed that LDH had an important clinical value in predicting MPNP. This study provides a potential serologic indicator for early diagnosis of MPNP.

## Introduction

*Mycoplasma pneumoniae* (MP) is a common pathogen that causes lower respiratory tract infections in children^1^ and cause up to 20 to 40% of community-acquired pneumonia^2^. In China, *Mycoplasma pneumoniae* pneumonia (MPP) accounts for about 32.4% of community-acquired pneumonia in children^3^. Necrotizing pneumonia (NP) is a disease characterized by inflammation and necrosis of lung tissue caused by pulmonary infection. In the early stages, the disease presents as consolidation of the lung, followed by liquefaction necrosis and the formation of multiple cystic or thin-walled cavities^4^. NP is usually caused by *Streptococcus pneumoniae* and *Staphylococcus aureus*^4^. In recent years, the incidence of *Mycoplasma pneumoniae* necrotizing pneumonia (MPNP), a specific form of necrotizing pneumonia caused by *Mycoplasma pneumoniae*, has been found to be on the rise, and MP is expected to become the main pathogen of NP^5–7^. MPNP is a severe complication of *MPP*, characterized by a prolonged and severe course of illness^2,6^. Patients may develop complications such as pneumothorax and bronchopleural fistula, which can even be life-threatening^8^. In view of the severity of NP and heavy burden of hospitalization, early prediction is essential for good recovery and prognosis.

Currently, contrast-enhanced chest CT is still the most sensitive modality for the diagnosis of NP^9^. Pulmonary consolidations present several weeks before cavities appear. It has been shown that the CT value of pulmonary lesions in NP was lower than that in non-NP and may help predict NP early^2^. However, in order to minimize radiation hazards, it is necessary to find new serological markers. Dan and colleagues investigated the potential of the peripheral blood neutrophil-to-lymphocyte ratio (NLR) to predict outcomes in patients with MPP. They found that Patients with a high NLR were more likely to develop NP and refractory Mycoplasma pneumoniae pneumonia (RMPP) and require intensive care, and had longer total fever duration, longer hospital stays, and higher hospitalization expenses than those with a low NLR^1^. The results suggested that the NLR can serve as a predictor of poor prognosis in patients with MPP and can predict the occurrence of NP, RMPP, and other poor outcomes. Xue’s^7^ research revealed that age, fever days, alanine transaminase (ALT), immunoglobulin M (IgM), complement C3, fibrinogen, dyspnea, and needing fiberoptic bronchoscopic alveolar lavage were independent risk factors for MPNP in children. Xue’s study also reported that serum lactate dehydrogenase (LDH) was higher than in the MPNP group were higher than those in the control group. Consistent with this result, Zhou’s et.al^2^ reported that MPP patients with NP might be easier to suffer from persistent fever, pulmonary consolidation, result in elevated LDH, WBC, CRP and disorder of cytokines. Paying close attention to these indicators may help in the early diagnosis of children suspected of MPNP.

Lactate dehydrogenase (LDH) is an enzyme involved in the anaerobic metabolism of the body, and it is widely distributed in red blood cells, myocardium, renal cells, hepatocytes and lung tissues. LDH is a pan-necrosis marker that can be released by cells undergoing primary or secondary necrosis^10^. When lung tissue is damaged, the permeability of cell membranes increases, resulting in the release of LDH into the bloodstream, which cause an increase of serum LDH^11^. During pneumonia, the inflammation and hypoxia in the lungs lead to increased serum LDH. In pediatric NP, the level of pleural fluid LDH is often > 1000 U/L and serum LDH is ≥ 353.5 U/L^4^. Paying attention to the high level of LDH may be useful in informing clinicians of the possibility of ongoing necrosis of the pulmonary parenchyma^12^. Therefore, investigation of the predictive value of LDH in MPNP in children and establishing an early prediction model for MPNP in children is crucial for diagnosis, treatment, and improving prognosis.

In this study, we explored the association strength between LDH continuous changes and MPNP using restricted cubic splines and evaluated the predictive value of developing MPNP in children with pneumonia caused by *Mycoplasma pneumoniae* and lung consolidation using decision curve analysis.

## Results

### General situation of two sets of related clinical factors

The clinical information of enrolled patients was shown in Table 1. There was no significant difference in age, gender, high fever, and wheezing between the consolidation group and the necrosis group (*P* > 0.05). While, the WBC count, PLT count, NE% were higher in necrosis group than consolidation group. Corresponding to this, the serum LDH, CRP, PCT, ESR, D-D, FIB, ALT, IgG, and IgM levels in necrosis group were significantly higher than consolidation group, as well as the differences in chest pain and dyspnea between the two groups were statistically significant (*P* < 0.05).

**Table 1:** Comparison of general conditions and related clinical factors between consolidation group and the necrosis group.

Clinical information	Consolidation group (N = 118)	Necrosis group (N = 128)	χ/T/Z value	*P*-value
Age	6.04 ± 2.22	6.53 ± 2.25		
Gender (male/female)	68/50	68/60	0.53	0.48
High fever (n, %)	114 (96.6)	128 (100)	−1.92	0.52
Chest pain (n, %)	3 (2.5)	59 (46.1)		< 0.01
Breathing (n, %)	4 (3.4)	1 (0.8)		0.213
Dyspnea (n, %)	2 (1.7)	32 (25)		< 0.01
WBC	8.79 ± 3.86	13.27 ± 5.99	−6.92	< 0.01
PLT	273.71 ± 105.797	389.96 ± 139.69	−6.92	< 0.01
Neutrophil ratio	62.66 ± 11.73	75.79 ± 12.43	−8.11	< 0.01
CRP	20.81 ± 22.65	76.04 ± 69.66	−8.41	< 0.01
PCT	0.21 ± 0.34	1.21 ± 4.47	−4.24	< 0.01
ESR	41.60 ± 21.03	64.34 ± 32.91	−5.01	< 0.01
D-D	0.77 ± 2.08	4.10 ± 3.75	−1.07	< 0.01
FIB	3.61 ± 1.17	4.18 ± 1.08	−5.17	< 0.01
ALT	23.687 ± 35.10	60.46 ± 120.26	−6.55	< 0.01
AST	30.86 ± 16.89	45.77 ± 45.64	−1.86	0.63
LDH	320.75 ± 131.86	569.12 ± 347.74	−8.65	< 0.01
IgE	388.97 ± 466.97	300.95 ± 411.38	1.09	0.27
IgA	1.41 ± 0.7	1.56 ± 0.766	−1.42	0.15
IgG	8.93 ± 2.33	11.25 ± 3.99	−4.060	< 0.01
IgM	1.73 ± 1.67	2.89 ± 1.61	−6.43	< 0.01

*WBC* white blood cells, *PLT* platelets, *NE%* percentage of neutrophils, *CRP* C-reactive protein, *PCT* pro calcitonin, *ESR* erythrocyte sedimentation rate, *D-D* d-dimer, fibrinogen, *ALT* alanine transaminase, *AST* aspartate aminotransferase, *LDH* lactate dehydrogenase, *IgE* immunoglobulin E, *IgA* immunoglobulin A, *IgG* immunoglobulin G, *IgM* immunoglobulin M.

### Correlation analysis of clinical factors and MPNP incidence

**Figure 1:**
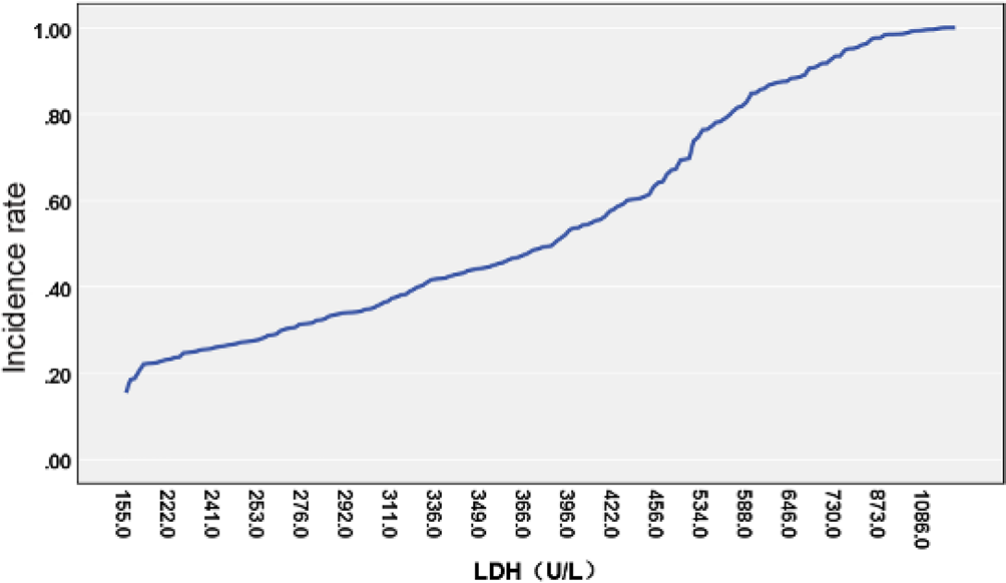
Correlation analysis of LDH and MPNP incidence.

The relationship between LDH values and the incidence of MPNP was calculated using LDH values as the horizontal axis and the probability of MPNP incidence as the vertical axis. The probability curve of LDH and MPNP incidence was plotted through single factor logistic regression, as shown in Fig. 1. The result shows that LDH values had corresponding MPNP incidence. Multivariate logistic regression analysis was then performed by univariate analysis with statistically significant indicators listed as independent variables and the incidence of MPNP as the dependent variable. The results suggested that LDH, PLT, DD and IgG were independent predictors of MPNP incidence (all *P* < 0.05), as shown in Table 2.

**Table 2:** Multivariate logistic regression analysis of MPNP incidence.

Variables	B	SE Wald χ two	P	OR	95% CI
PLT	0.001	7.352	0.007	1.006	1.002–1.010
DD	0.231	3.997	0.046	1.260	1.005–1.579
AST	−0.020	3.331	0.068	0.980	0.960–1.001
LDH	0.006	8.978	0.003	1.006	1.002–1.010
IgG	0.274	6.565	0.010	1.315	1.067–1.622
Chest pain	1.221	2.546	0.111	3.390	0.757–15.184
Dyspnea	3.482	5.500	7.019	32.538	1.772–597.554
Constant	−6.920	23.051	0.001	0.001	

### The predictive value of LDH for the incidence of MPNP

**Figure 2:**
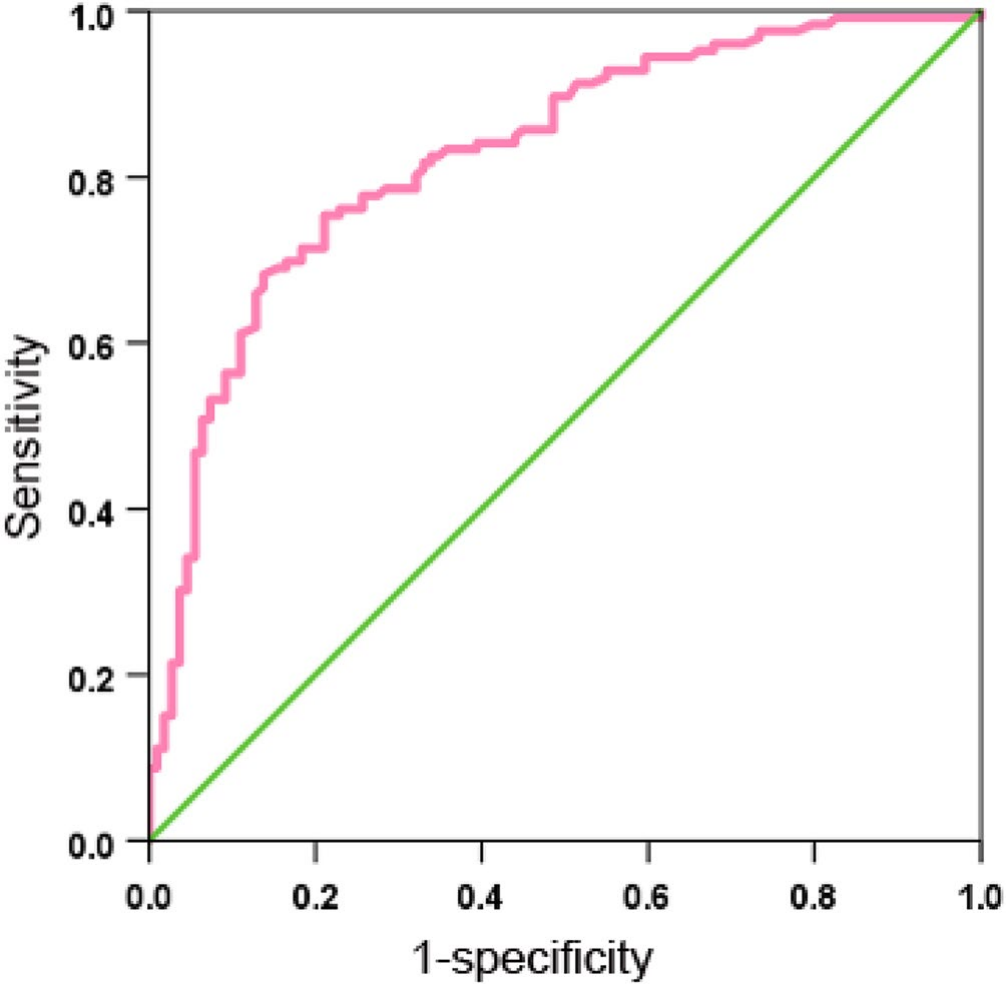
ROC curve of LDH predicting MPNP incidence.

Then, we evaluated the predictive value of LDH for the incidence of MPNP. Taking LDH as the predictor, sensitivity as the ordinate and 1-specificity as the abscissa, the calculated area under the ROC curve 0.827 (95% CI: 0.774–0.880). When LDH is 393.0 U/L, the Jordan index is the highest, at 0.545. Therefore, setting LDH at 393.0 U/L as the optimal cutoff value, the sensitivity and specificity of prediction are 68.3% and 86.2%, respectively. When LDH > 393.0 U/L, as LDH increases, the risk of developing necrotizing pneumonia in children with Mycoplasma pneumoniae pneumonia and pulmonary consolidation significantly increases (Fig. 2).

### The dose–response analysis of the correlation between LDH and MPNP

**Figure 3:**
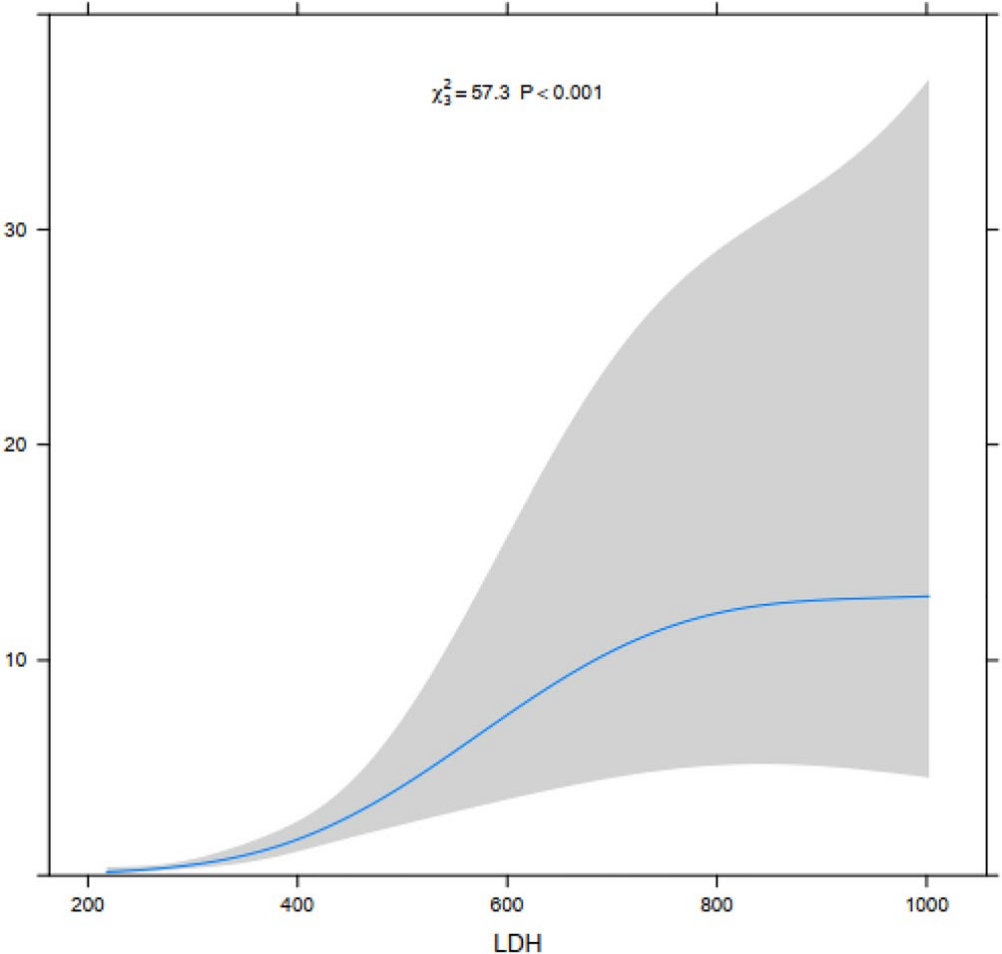
The dose–response relationship between LDH and MPNP based on the restricted cubic spline model. The continuous change of LDH and the intensity of the correlation between MPNP exhibited a nonlinear dose–response relationship.

The dose–response analysis of the correlation strength between LDH and MPNP was conducted using the optimal cutoff value of LDH at 393.0 U/L as the reference value. The restricted cubic spline method combining spline function and logistic regression was used to analyze the dose–response relationship between LDH and MPNP. The horizontal axis represents the continuous change of LDH, the vertical axis represents the corresponding predicted value (OR), and the shaded part represents 95% CI. Our study suggested that the correlation between continuous changes in LDH and MPNP showed a non-linear dose–response relationship (P < 0.01). The results showed a significantly positive correlation between LDH and the incidence of MPNP. With the LDH increasing especially when LDH > 393.0 UL the risk of MPNP disease significantly increased (Fig. 3).

### Decision curve analysis (DCA) of LDH and MPNP

**Figure 4:**
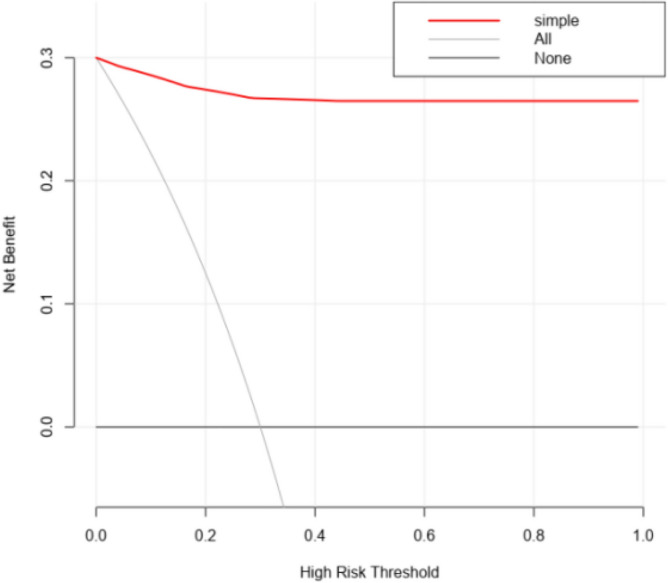
Decision curve analysis of LDH.

The sensitivity, specificity, and area under the ROC curve reflects the diagnostic accuracy of the model. However, they lack consideration of the clinical effectiveness of a particular model. The advantage of decision curve analysis lies in its integration of patient or decision-maker preferences into the analysis. In this study, the DCA curve was draw with the net profit rate as the vertical axis and the high-risk threshold as the horizontal axis (Fig. 4). The high-risk threshold is set to (0, 1). When the high-risk threshold is > 0 (the incidence risk value corresponding to LDH value can be obtained from Fig. 1), the net benefit rate is greater than 0 (actually > 0.25), which is clinically significant. (Fig. 4).

## Discussion

*Mycoplasma pneumoniae* is an important pathogen of children's community acquired pneumonia. The incidence rate of MPP is increasing, with worldwide incidence of 8·61% from 2017 to 2020^13^. Although nonpharmacologic interventions targeting COVID-19 greatly reduced *Mycoplasma pneumococcal* infection between 2020 and 2023^14^, however, if *Mycoplasma pneumococcal* infections resurge, it could affect a world population that has not been exposed to *Mycoplasma pneumoniae* in the past 3 years^15^ and result in an increase in rare severe diseases and extrapulmonary manifestations^16^. Early prediction is essential for good recovery and prognosis of *Mycoplasma pneumoniae* and is an effective means of dealing effectively with *Mycoplasma pneumoniae* infection.

LDH is present in the cytoplasm of cells. When cells are infected, cytoplasmic membrane permeability increases and LDH is released into the bloodstream. Therefore, LDH can be used as a serologic marker of disease. Pulmonary complications in children with severe *M. pneumoniae* pneumonia are associated with higher levels of LDH and LDH to albumin ratio^8,17,18^. The level of LDH in children with MPNP [428.50 (319.25–598.25)] were higher than children with MPP [351.00 (297.25–470.23)]^7^. It may because MPP patients with NP might be easier to suffer from persistent fever, pulmonary consolidation, result in elevated WBC, CRP, LDH and disorder of cytokines^2^.

However, the cutoff of LDH values were inconsistent across reports. Chen’s study revealed that the level of pleural fluid LDH is often > 1000 U/L in NP among children, while serum LDH is ≥ 353.5 U/L^9^. Thus, serum LDH ≥ 353.5 U/L and/or pleural fluid LDH > 1000 U/L), NP would be more suspicious. Zhang’s study revealed that LDH in 428.50 (319.25–598.25) U/L in MPNP and 351.00 (297.25–470.23) in control group^7^. Luo and colleagues’ study reported that the LDH level in serum of necrotizing pneumonia group is 336.00 [274.00, 412.00] U/L and 316.00 [271.75, 365.50] U/L in non-necrotizing pneumonia group^19^. While Zhou’s study^2^ and Yang’s study^20^ reported that LDH was 646 (393–971) U/L and 805.0 (423.7–1029.5) U/L in MPNP group, and 494 (388–693) and 414.0 (299.9–540.6) in non-MPNP. These may be closely related to sample size, sample population, and age.

This study indicated that LDH was an independent factor in the incidence of MPNP in children. The area under the ROC curve was 0.827 indicating that the accuracy of LDH in predicting MPNP in children was consistent with previous research^21^. The cutoff value of LDH in this study was 393.0 U/L, and the sensitivity and specificity of prediction were 68.3% and 86.2%, respectively. Previous study^21^ has calculated the correlation strength between LDH and MPNP in traditional logistic models regardless of the trajectory of changes in LDH continuity and MPNP correlation strength. Artificially segmenting LDH for research may not only result in loss of information but may also result in inaccurate results. Our study matched the baseline data of children by the propensity score matching method which avoided bias in baseline data. We also found that LDH was an independent predictive factor for the incidence of MPNP through multiple logistic regression analysis. Dose response analysis was used to observe the prediction of different changes in LDH on the risk of MPNP incidence, and decision curve analysis was used to evaluate its predictive value. However, the present study still has some limitations, such as the lack of external controls, which prevents the validation of the scalability of the established models. Therefore, future prospective experiments with the currently established indicators are necessary to understand the stability of the model.

In summary, LDH is an independent predictor of the incidence of MPNP in children and has important value in predicting MPNP. It has a significant nonlinear dose–response relationship with MPNP incidence. The risk of MPNP incidence significantly increases with the increasing LDH especially when LDH > 393.0 U/L.

## Methods

### Study population.

This is a retrospective study and focuses on 4899 children with MPP who were hospitalized at the Affiliated Children's Hospital of Zhengzhou University from January 2019 to June 2022. All selected children must meet the following criteria: ① 1–14 years old; ② the diagnostic of MPP was according to Zhu Futang Practical Pediatrics criteria, which with fever and cough as the main clinical manifestations, chest X-ray or chest CT indicating pneumonia, single MP antibody titer ≥ 1:160, MP antibody titer increased or decreased by 4 times or more in the recovery and acute stages, single test positive for MP-IgM and positive for MP DNA in nasopharyngeal swab or alveolar lavage fluid; ③ Duration of disease at admission: 1–3 weeks. Within 24 h of admission, routine blood tests, percentage of neutrophil, C-reactive protein (CRP), lactate dehydrogenase (LDH), d-dimer, liver function and other related tests are performed. Children with previous history of bronchial asthma, recurrent respiratory infections, primary or secondary immune dysfunction, congenital heart disease, and those who are infected with other pathogens, as well as those with incomplete medical records were excluded.

### MPNP inclusion criteria

Chest CT shows early bilateral or unilateral lung consolidation, followed by liquefaction and necrosis in the consolidation area, forming multiple thin-walled cavities or cystic shadows that can fuse into large cavities, meeting the diagnostic criteria for necrotizing pneumonia.

### Inclusion criteria for consolidation group

Chest imaging shows infiltration in more than one lung segment or lobe, and no progression to necrotizing pneumonia. Chest CT was carried out by using Brilliance CT 64 Slice [Philips Medical Systems (Cleveland) Inc.]. Patients with pulmonary tuberculosis, congenital lung cysts and lung abscesses were excluded. Considering that liver injury affects serum LDH levels, patients with diseases such as viral hepatitis and metabolic diseases that affecting the liver were excluded. Diagnostic criteria for liver injury: ALT > 3 times the upper limit of normal value (ULN), and the ratio of ALT measured value/ALT normal value upper limit to ALP measured value/ALP normal value upper limit is ≥ 5.

This study has been approved by Ethics Committee of Children's Hospital of Henan Province and all methods were performed in accordance with the relevant guidelines and regulations. Informed consent from a parent and/or legal guardian of the minor participant was taken for study participation. The hypothesis of this study was that LDH effectively differentiate between patients and healthy controls, and the area under the ROC curve for LDH is greater than 0.5. Based on the previous literature it was known that the area under the ROC curve for LDH was 0.836, and the sample size was estimated using the PASS15 at α = 0.05 (biparietal), β = 0.1, and the ratio of the groups was 1:1. It was found that a minimum of 13 patients and 13 controls, totaling 26, needed to be included. Considering a 10% loss to follow-up rate, the study included 15 patients and 15 controls, totaling 30. Among the remaining 3250 children with MP, the propensity score method was used to match them at a 1:2 ratio, as each MPNP group individual was matched with 2 non MPNP individuals with the most similar propensity score values. Finally, 128 children with MPNP met the criteria for necrotic group, and 118 children with MPP combined with pulmonary consolidation were met the criteria for the consolidation group.

### Data collection

We obtained the MPP patients’ basic data, such as age, gender, course of disease at admission and bodyweight by retrospectively review. We used propensity score matching to match the two groups of patients to obtain a covariate-balanced sample between the two groups, and then collected clinical presentations, laboratory data and imaging findings from the sample.

The clinical manifestations mainly include high fever, shortness of breath, fine wet rales, wheezing, liver damage, and skin damage. Laboratory tests mainly include white blood cell (WBC) count, platelet (PLT) count, neutrophil percentage (NE%), C-reactive protein (CRP), Calcitonin (PCT), alanine transaminase (ALT), aspartate aminotransferase (AST), LDH content, D-dimer, fibrinogen, IgE, IgA, IgG and IgM. The imaging examination results mainly include the presence or absence of pleural effusion, lung consolidation, atelectasis, and pulmonary necrosis.

### Statistical analysis

The statistical analysis was performed using SPSS 24.0 and R 3.60. The distribution of data in all groups was tested for normality of data distribution by Kolmogorov–Smirnov test, and data satisfying normal distribution were evaluated by t-test, expressed as x ± SD (mean ± standard deviation). For non-normally distributed data, Mann–Whitney rank sum test was used, expressed as median (quartile interval) [M (JOR)]. Comparisons between two groups of quantitative data were expressed as (%) by chi-square (χ^2^) test or Fisher's exact probability method. Receiver operating characteristic (ROC) curve analysis was used to evaluate the value of LDH in the prediction and diagnosis of MPNP. *P* values were calculated using the two-tailed test. *P* < 0.05 was considered statistically significant.

Firstly, compare the differences of indicators between the necrosis group and the consolidation group, and further conduct logistic regression analysis. If the independent variable has a *P* < 0.1 in the results of a one-way logistic regression analysis, then it should be included in the multivariate logistic regression analysis. The independent variables were screened using the forward method and differences were considered statistically significant at *P* < 0.05. The probability curve of LDH and MPNP incidence was draw using logistic regression model. LDH level were used as the prediction indicator. Receiver operating characteristics were plotted with sensitivity as the vertical coordinate and 1-specificity as the horizontal coordinate. At the same time, we find the best cut-off value of LDH by using the Youden's J statistic method and evaluated the relationship between LDH and MPNP incidence OR values using a restricted cubic spline function. The nonlinear relationship between the independent variable and the dependent variable were analyzed by the restricted cubic spline method that combines the spline function and logistic regression. With the best cut-off value of LDH as the reference value, the restricted cubic spline method was used to analyze the dose response relationship between LDH and MPNP. LDH was a continuous variable and four nodes (P5, P35, P65, P95) were selected based on their quantitative distributions and restricted cubic spline graphs were plotted in R 3.60 software. Finally, decision curve analysis (DCA) on LDH was performed. Decision curve analysis is a statistical method for evaluating clinical predictive models, diagnostic experiments, and molecular markers. Using the net benefit rate as the vertical axis and the high-risk threshold as the horizontal axis, DCA curves were plotted to comprehensively analyze whether LDH can generate effective clinical value in clinical application decisions with different probabilities.

## Data Availability

The datasets used and analyzed in the present study is available from the corresponding author on reasonable request.
